# Thirty years of home mechanical ventilation in children: escalating need for pediatric intensive care beds

**DOI:** 10.1007/s00134-012-2545-9

**Published:** 2012-04-05

**Authors:** Fleur M. Paulides, Frans B. Plötz, Laura P. Verweij-van den Oudenrijn, Josephus P. J. van Gestel, Mike J. Kampelmacher

**Affiliations:** 1 Department of Pediatric Intensive Care, VU Medical Center, Amsterdam, The Netherlands; 2 Center for Home Mechanical Ventilation, D01.225, University Medical Center Utrecht, P.O. Box 85500, 3508 GA Utrecht, The Netherlands; 3 Department of Pediatric Intensive Care, University Medical Center Utrecht, Utrecht, The Netherlands; 4 Department of Pediatrics, Tergooi Hospitals, Blaricum, The Netherlands

**Keywords:** Chronic respiratory failure, Home mechanical ventilation, Children, Non-invasive mechanical ventilation, Intensive care beds, pediatric intensive care unit

## Abstract

**Purpose:**

To describe trends in pediatric home mechanical ventilation (HMV) and their impact on the use of pediatric intensive care unit (PICU) beds.

**Methods:**

Review of all children who had started HMV in a single center for HMV.

**Results:**

Between 1979 and 2009, HMV was started in 197 patients [100 (51 %) with invasive and 97 with noninvasive ventilation], with a median age of 14.7 (range 0.5–17.9) years. Most patients (77 %) were males with a neuromuscular disorder (66 %). The number of children receiving HMV increased from 8 in the 1979–1988 period to 122 in the 1999–2008 period. This increase occurred foremost in patients aged 0–5 years and was accompanied by a sharp rise in the use of PICU beds. In 150 patients (76 %), HMV was initiated on an ICU with a total of 12,440 admission days, of which 10,385 days (83 %) could be attributed to 67 patients who started non-electively with invasive HMV. Of the latter, 52 patients had been admitted to a PICU with a total of 9,335 admission days. At the end of the study, 134 patients (68 %) were still being ventilated, 43 patients (22 %) had died, 11 patients (6 %) were weaned from HMV, 4 patients (2 %) did not want to continue HMV and 5 patients (3 %) were lost to follow-up.

**Conclusions:**

Over time, there was an impressive increase in the application of HMV in children. This increase was most obvious in the youngest age group with invasive HMV, and these children had very long stays in the PICU.

## Introduction

In patients with chronic respiratory failure the aims of home mechanical ventilation (HMV) include prolonging life, while maintaining or improving the quality of life, and reducing morbidity [1–6]. In the past decades the number of adults that benefitted from HMV seems to have increased steadily. This growth may be explained by the immense success of HMV and thus a greater awareness among physicians and patients; improvements in technology and medical care; and by an increased patient autonomy and a different moral-ethical attitude with respect to treatment decisions. In agreement with the trend in adults, we got the impression that HMV was also being applied in children more often, at an increasingly younger age, and also for less ‘classical’ diseases. Literature confirming this is, however, sparse [7–10]. Given these changes, we were interested in their possible impact on the use of pediatric intensive care beds. With the improvements that had been realized over the years with regard to discharge planning, we were particularly interested in the length of stay following the initiation of HMV in the pediatric intensive care unit (PICU). The goals of this study were therefore to describe [1] the experiences and changes in applying HMV in children and [2] the impact of these changes on the use of PICU beds.

## Patients and methods

The study was designed as a retrospective, single-center observational study in the Center for HMV Utrecht, which is part of the University Medical Center Utrecht and one of the four HMV Centers in the Netherlands. The need for institutional review board approval was waived.

### Patients and setting

All patients who had started HMV before the age of 18 before 1 January 2009 were included. The standard operating procedure for HMV in our Center is that children undergo an intake, either during a visit at the outpatient clinic or in the hospital where they have been admitted. HMV can then be initiated after all required information has been given and with mutual and thoughtful agreement of the patient, family and medical team of the Center for HMV. Generally, HMV is then initiated when patients have clinical manifestations of (nocturnal) hypoventilation together with (nocturnal) hypercapnia (PaCO_2_ > 45.0 mmHg or 6.0 kPa). The presence of hypercapnia could be established in a (P)ICU, a hospital ward, or a specially designated respiratory care unit (which started off in 1997), mainly depending on the child’s age and clinical circumstances. The setup of HMV was regarded as elective if clinically stable patients had been admitted on behalf of the Center for HMV because of suspected nocturnal or proven (diurnal) ventilatory failure. In patients who were unknown to the Center for HMV before admission to an ICU or in patients known to the Center for HMV who had an unforeseen admission to an ICU, the initiation of HMV was regarded as non-elective.

Setting up HMV was a joint action of the nurses and physicians of the Center for HMV and the ward where the patient was admitted. Discharge planning was a multidisciplinary effort, which was coordinated by a nurse of the Center for HMV. Funding, training and the provision of equipment never caused a problem, but housing, family issues and particularly recruitment of competent professionals sometimes did. Following discharge, the Center for HMV took care of patients by supplying all necessary equipment and materials, regular and intermittent home visits, continuous accessibility and continuous education of all persons involved in the care of these patients. In case of medical problems not related to HMV, patients were seen by their general practitioner and primarily referred to a local hospital, if needed.

### Data collection and definitions

Demographic and clinical data of patients were retrieved from their medical records. The underlying diseases were divided into four categories: neuromuscular disorders (like spinal muscular atrophy, Duchenne muscular dystrophy), central nervous system diseases (like Arnold Chiari malformation, congenital central hypoventilation syndrome), airway diseases (like bronchopulmonary dysplasia, cystic fibrosis) and miscellaneous diseases (like congenital scoliosis). The children were divided into three age groups (0–5, 6–11 and 12–17 years) and 3 decades (1979–1988, 1989–1998 and 1999–2008) in which their HMV was initiated.

Specific data from PICU admissions were noted: day of admission to the PICU, the day that ventilation with a home care ventilator was started and the day patients were discharged from the PICU. The numbers of days in the PICU before and following initiation of ventilation with a home care ventilator are referred to as the pre- and post-HMV period, respectively.

## Results

Between 1 January 1979 and 1 January 2009, 197 children had started with HMV (Table 1). HMV was started at a median age of 14.7 years (range 0.5–17.9 years). Most patients (63 %) were between 12 and 18 years when HMV was started, and most of them were males with a neuromuscular disorder. Invasive HMV was started in 100 patients. We observed that over the 3 decades the percentage of invasive ventilation decreased from 100 to 39 %. In 108 patients (55 %) HMV was started non-electively. This occurred most often in the youngest age group, and most of these patients required invasive mechanical ventilation. Patients in the youngest age group had various underlying diseases (Table 1).

**Table 1:** Characteristics of all children who started home mechanical ventilation (HMV) before the age of 18 years, between 1 January 1979 and 1 January 2009

	Age of HMV onset			
	All patients	0–5 years	6–11 years	12–17 years
	*n* = 197	*n* = 41	*n* = 32	*n* = 124
Demographic data				
Median age (years) onset HMV	14.7 (0.5–17.9)	1.5 (0.5–5.5)	9.5 (6–12)	15.9 (12–17.9)
Male/female (%)	77/23	63/37	66/34	85/15
Median time of follow-up (years)	5.6 (0–29.8)	2.9 (0–26.2)	7.2 (0.5–29.8)	6.3 (0–25.2)
Underlying diseases (*n*, %)				
Neuromuscular disorders	130 (66)	14 (34)	18 (56)	98 (79)
Central nervous system disorders	33 (17)	12 (29)	8 (25)	13 (10)
Chronic pulmonary or airway diseases	13 (6)	7 (17)	1 (3)	5 (4)
Miscellaneous	21 (11)	8 (20)	5 (16)	8 (7)
Indication (*n*, %)				
Non-elective (all patients)	108/197 (55)^a^	35/41 (85)	13/32 (41)	60/124 (48)
1979–1988	6/8 (75)	1/1 (100)	1/2 (50)	4/5 (80)
1989–1998	34/67 (51)	8/10 (80)	6/10 (60)	20/47 (43)
1999–2008	68/122 (56)	26/30 (87)	6/20 (30)	36/72 (50)
Start HMV, type of support (*n*, %)				
Invasive ventilation (all patients)	100/197 (51)	31/41 (76)	11/32 (34)	58/124 (47)
1979–1988	8/8 (100)	1/1 (100)	2/2 (100)	5/5 (100)
1989–1998	45/67 (67)	8/10 (80)	7/10 (70)	30/47 (64)
1999–2008	47/122 (39)	22/30 (73)	2/20 (10)	23/72 (32)
Ventilator dependency at end of study	*n* = 134	*n* = 29	*n* = 25	*n* = 80
Nocturnal ventilation only (%)	46	55	64	36
Ventilation during 8–20 h/day (%)	17	28	20	13
Ventilation 20–24 h/day (%)	37	17	16	51

^a^Number and percentage (between brackets) of relevant cases per (sub) group for that particular decennium and age group

**Fig. 1:**
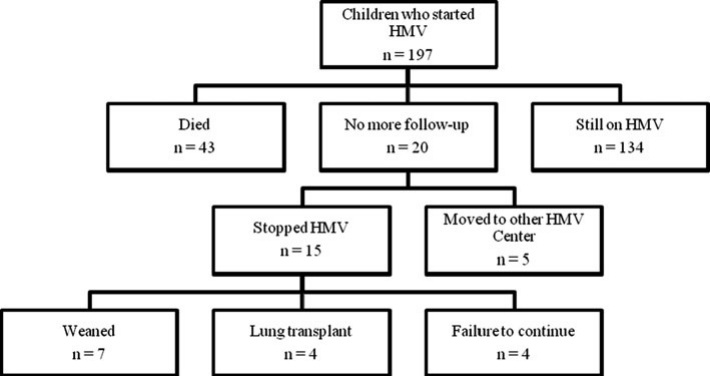
Flow diagram summarizing the outcome of children who started HMV before the age of 18 years between 1979 and 2009

At the end of the study, patients had been on HMV for a median of 5.6 years (range 0–29.8 years). On 1 January 2009, 134 patients (68 %) were still actively ventilated, 43 patients (22 %) had died, 11 patients (6 %) were weaned from HMV, including 4 patients with cystic fibrosis who underwent a lung transplant, 4 patients (2 %) did not want to continue HMV and 5 patients (3 %) were lost to follow-up (Fig. 1). The characteristics of the 43 patients who died are presented in Table 2. The most common causes of death were cardiac (*n* = 15; mostly Duchenne patients) or chest infection (*n* = 5). Two patients died after accidental decannulation.

**Table 2:** Characteristics of the patients who died

	Deceased patients		
	All patients	Non-invasive	Invasive
	*n* = 43	*n* = 14	*n* = 29
Demographic data			
Age (years) at start HMV (median, range)	13.2 (0.7–17.6)	15.2 (12.2–16.7)	12.2 (0.7–17.6)
Age (years) of death (median, range)	20.2 (1.2–37.1)	18.2 (13.9–29.9)	21.2 (1.2–37.1)
Time of ventilation in years (median, range)	7.5 (0.3–20.7)	4.2 (1.1–13.1)	8.8 (0.3–20.7)
Number of admission days post HMV (median, range)	28.1 (2–254)	9.9 (2–21)	38.2 (2–254)
Start with HMV (*n*)			
1979–1988	1	0	1
1989–1998	27	6	21
1999–2008	15	8	7
Underlying diseases (*n*)			
Neuromuscular disorders	36	13	23
Central nervous system conditions	3	0	3
Chronic pulmonary or airway diseases	2	1	1
Miscellaneous	2	0	2
Location of death			
Home	27	7	20
Hospital	16	7	9

### Changes over time

**Fig. 2:**
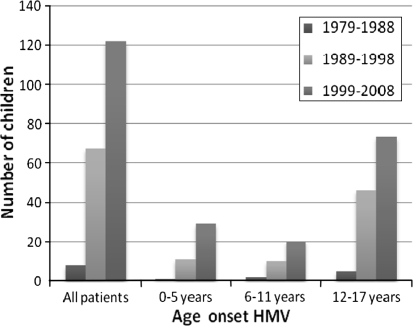
Number of children that started home mechanical ventilation (HMV) depending on decade and age group

Over the years, there was a substantial increase in the number of children receiving HMV (Fig. 2). During the 1979–1988 period, only 8 patients received HMV (all invasive, see Table 1), and this increased to 122 in the 1999–2008 period [of whom 47 (39 %) started with invasive HMV, see Table 1]. Comparing the different age groups with respect to receiving HMV, we noticed a 1.5-fold increase in older children (12–17 years), a 2-fold increase in children aged 6–11 years and a 3-fold increase in the youngest children (0–5 years) over the last 10 years in comparison to the preceding 10 years, respectively (Fig. 2). Over the years, the proportion of patients who started with HMV non-electively slightly decreased from 75 % (6 out of 8 patients) in the 1979–1988 period to 56 % (68 out of 122 patients) in the 1999–2008 period, respectively.

### PICU length of stay

In 150 patients (76 %), HMV was initiated in an ICU. The total number of admission days was 12,440. The majority of the admission days, namely 10,385 (83 %), could be attributed to 67 patients who started invasive HMV non-electively. Of these 67 patients, 52 had been admitted to a PICU with a total number of 9,335 admission days. Considering this observation, a sub-analysis was performed of the PICU length of stay in this last category of patients: it was further divided in pre-HMV and post-HMV days over the 3 decades. The results of this analysis are given in Table 3. The total PICU length of stay increased considerably over the 3 decades. This was attributed almost exclusively to the youngest age group. The length of stay per patient decreased in the last decade; therefore, the rise in total PICU length of stay is chiefly explained by an increase in the number of young children starting with non-elective, invasive HMV.

**Table 3:** The number of total PICU admission days as well as the number of admission days before (pre-HMV) and following (post-HMV) the day that invasive ventilation was started non-electively on a home care ventilator, depending on decade and age group

	All patients	0–5 years	6–11 years	12–17 years
1979–1988				
All patients (*n*)	2	1	1	0
Total number of days	622	222	400	0
Pre-HMV	506	206	300	0
Post HMV	116	16	100	0
1989–1998				
All patients (*n*)	18	6	5	7
Total number of days	2,770	2,032	188	550
Pre-HMV	1,848	1,349	113	386
Post HMV	922	683	75	164
1999–2008				
All patients (*n*)	32	20	1	11
Total number of days	5,943	5,050	37	856
Pre-HMV	4,311	3,699	20	592
Post HMV	1,632	1,351	17	264

## Discussion

This observational study of 197 children on HMV represents one of the largest pediatric cohorts reported to date, and the first to describe experiences and trends in applying HMV over a period of 3 decades. The most striking change over time is the steep rise in the number of children in whom HMV was being applied, particularly during the last decade and in the youngest age group. Especially these children had very long stays in the PICU, both before and after HMV was initiated.

There are several explanations for the increase in the application of HMV in children. Presumably, the success of HMV in adults has led to a greater awareness among physicians involved in the care for children. Improvements in medical health care, increased patient autonomy and changing moral-ethical attitudes with respect to treatment decisions may explain the growing number of children admitted to and kept alive in the PICU [11]. Improvements in technology have made HMV not only an alternative for death, but also succeeded in improving the quality of life for most of these children; consequently children are nowadays being referred for HMV more frequently [12].

We found an escalating need for PICU beds, especially for the youngest children (Table 3), and specifically these children consumed the most PICU admission days. The retrospective nature of our study limited an in-depth investigation of the reasons for these prolonged stays. The pre-HMV length of stay probably reflects the complexity of the medical condition in young children: most of them were admitted after an acute deterioration of their respiratory condition, often before the diagnosis or prognosis of the underlying disorder was made. This is underscored by the high incidence of non-elective initiation of HMV in this group. We have the impression that the most time post-initiation of HMV was required for fine-tuning ventilator settings, training parents and organizing professional home care. In the youngest children, with often less stable respiratory conditions, these steps are often more difficult and time-consuming than in older children.

The long post-HMV period in the youngest age group particularly raises our concern. Though lower per patient than in the UK [13] and the US [14], it amounted to a total of 1,351 post-HMV PICU days in the last decade, which means blockade of one PICU bed for about 4 months for each year in the last decade. During the last decade, a discharge coordinator was introduced in our Center for HMV, and efforts have been made to streamline the discharge process in order to shorten PICU admission. As shown in Table 3, this resulted in a decrease of post-HMV days per patient compared to the previous decade, but despite this, the total PICU length of stay rose considerably because of an increase in young patients starting with HMV. Since it is likely that this trend is going to continue, the need for PICU beds will probably continue to rise as well. It could be worthwhile to establish designated units outside the PICU for clinically stable children requiring HMV [10]. Such a setting, which is less stressful and more focused on rehabilitation than on acute care, might not only be a better but also a cheaper and safe alternative [15–18].

The amount of literature on trends in the application of HMV in children is limited. We are aware of only one other study covering the same time span, but this study contained both children with and without HMV and had different end points [8]. Other studies covered a shorter time span [10, 19, 20] or a single time point [7, 9, 21, 22], contained children with either invasive [13, 20] or noninvasive HMV [23], or were mainly focused on survival [15] or hospital admission days [14]. In comparison to other studies, invasive HMV was used with a comparable frequency (38 %) as in Massachusetts (49 %) or Italy (41 %), but markedly more frequently than in Australia (22 %), the UK (23 %) or Turkey (32 %) [7–9, 21, 24]. Invasive HMV is our first choice for the youngest children as they have already been tracheotomized during their stay in the PICU and need to keep a tracheostomy for the time being to evacuate airway secretions. Moreover, noninvasive ventilation may cause mid-facial retrusion if applied in early childhood and is often poorly tolerated, particularly when applied for 24 h per day [25]. Improvements in interfaces for noninvasive HMV and new developments such as mechanical insufflation/exsufflation have, however, caused a decline in tracheostomal ventilation, also for very young patients [26, 27].

At the end of our study, 11 children (6 %) were eventually weaned off of HMV. In other studies weaning varied from 9 to 39 % [8, 10, 13, 14, 24]. Mortality was considerable with 22 %, but nearly always related to the underlying disorder, and comparable to the mortality of 7–32 % found in other cohorts [8, 10, 13, 14, 20, 24]. The observation that most patients who died had used HMV for several years strongly suggests that HMV prolonged their lives.

Our retrospective study has several limitations. Being a single center study, our results may not be automatically applicable to other centers. However, the four Centers for HMV in the Netherlands have always been comparable with respect to their procedures, and we feel that our results reflect the Dutch situation. As HMV in the Netherlands is well organized, it is unclear to what extent our findings can be extrapolated to patients in other countries. Given the number of patients and time span involved we consider our results relevant for providing trends with regard to HMV in children and, thereby, for comparison with other studies.

In conclusion, in the past 3 decades the number of children, and particularly those aged 0–5 years, who received HMV in our center increased substantially. This was associated with an escalating need for pediatric intensive care beds. If this trend continues, the establishment of specialized facilities outside the PICU for clinically stable children requiring HMV should be seriously considered.

## References

[1] Ward S, Chatwin M, Heather S, Simonds AK (2005). Randomised controlled trial of non-invasive ventilation (NIV) for nocturnal hypoventilation in neuromuscular and chest wall disease patients with daytime normocapnia. Thorax.

[2] Dohna-Schwake C, Podlewski P, Voit T, Mellies U (2008). Non-invasive ventilation reduces respiratory tract infections in children with neuromuscular disorders. Pediatr Pulmonol.

[3] Windisch W (2008). Impact of home mechanical ventilation on health-related quality of life. Eur Respir J.

[4] Tzeng AC, Bach JR (2000). Prevention of pulmonary morbidity for patients with neuromuscular disease. Chest.

[5] Jeppesen J, Green A, Steffensen BF, Rahbek J (2003). The Duchenne muscular dystrophy population in Denmark, 1977–2001: prevalence, incidence and survival in relation to the introduction of ventilator use. Neuromusc Disord.

[6] Windisch W, Walterspacher S, Siemon K, Geiseler J, Sitter H (2010). Guidelines for non-invasive and invasive mechanical ventilation for treatment of chronic respiratory failure. Published by the German Society for Pneumology (DGP). Pneumologie.

[7] Graham RJ, Fleegler EW, Robinson WM (2007). Chronic ventilator need in the community: a 2005 pediatric census of Massachusetts. Pediatrics.

[8] Tibballs J, Henning R, Robertson CF, Massie J, Hochmann M, Carter B, Osborne A, Stephens RA, Scoble M, Jones S, White J, Bryan D (2010). A home respiratory support programme for children by parents and layperson carers. J Pediatr Child Health.

[9] Wallis C, Paton JY, Beaton S, Jardine E (2011). Children on long-term ventilatory support: 10 years of progress. Arch Dis Child.

[10] Edwards EA, Hsiao K, Nixon GM (2005). Paediatric home ventilatory support: the Auckland experience. J Pediatr Child Health.

[11] Dyer C (2006). Judge rules that baby boy should not be allowed to die. BMJ.

[12] Chatwin M, Bush A, Simonds AK (2011). Outcome of goal-directed non-invasive ventilation and mechanical insufflation/exsufflation in spinal muscular atrophy type I. Arch Dis Child.

[13] Edwards EA, O’Toole M, Wallis C (2004). Sending children home on tracheostomy dependent ventilation: pitfalls and outcomes. Arch Dis Child.

[14] De Witt PK, Jansen MT, Davidson Ward SL, Keens TG (1993). Obstacles to discharge of ventilator-assisted children from the hospital to home. Chest.

[15] Edwards JD, Kun SS, Keens TG (2010). Outcomes and causes of death in children on home mechanical ventilation via tracheostomy: an institutional and literature review. J Pediatr.

[16] Ambrosio IU, Woo MS, Jansen MT, Keens TG (1998). Safety of hospitalized ventilator-dependent children outside of the intensive care unit. Pediatrics.

[17] MacIntyre NR, Epstein SK, Carson S, Scheinhorn D, Christopher K, Muldoon S (2005). Management of patients requiring prolonged mechanical ventilation: report of a NAMDRC consensus conference. Chest.

[18] Dasgupta A, Rice R, Mascha E, Litaker D, Stoller JK (1999). Four-year experience with a unit for long-term ventilation (respiratory special care unit) at the Cleveland Clinic Foundation. Chest.

[19] Appierto L, Cori M, Bianchi R, Onofri A, Catena S, Ferrari M, Villani A (2002). Home care for chronic respiratory failure in children: 15 years experience. Pediatr Anaesthesia.

[20] Gowans M, Keenan HT, Bratton SL (2007). The population prevalence of children receiving invasive home ventilation in Utah. Pediatr Pulmonol.

[21] Racca F, Berta G, Sequi M, Bignamini E, Capello E, Cutrera R, Ottonello G, Ranieri VM, Salvo I, Testa R, Wolfler A, Bonati M (2011). Long-term home ventilation of children in Italy: a national survey. Pediatr Pulmonol.

[22] Kamm M, Burger R, Rimensberger P, Knoblauch A, Hammer J (2001). Survey of children supported by long-term mechanical ventilation in Switzerland. Swiss Med Wkly.

[23] Fauroux B, Boffa C, Desguerre I, Estournet B, Trang H (2003). Long-term noninvasive mechanical ventilation for children at home: a national survey. Pediatric Pulmonol.

[24] Oktem S, Ersu R, Uyan ZS, Cakir E, Karakoc F, Karadag B, Kiyan G, Dagli E (2008). Home ventilation for children with chronic respiratory failure in Istanbul. Respiration.

[25] Fauroux B, Lavis JF, Nicot F, Picard A, Boelle PY, Clément A, Vazquez MP (2005). Facial side effects during noninvasive positive pressure ventilation in children. Intensive Care Med.

[26] Leboulanger N, Picard A, Soupre V, Aubertin G, Denoyelle F, Galliani E, Roger G, Garabedian EN, Fauroux B (2010). Physiological and clinical benefits of noninvasive respiratory support in infants with Pierre Robin sequence. Pediatrics.

[27] Fauroux B, Leboulanger N, Roger G, Denoyelle F, Picard A, Garabedian EN, Aubertin G, Clément A (2010). Noninvasive positive-pressure ventilation avoids recannulation and facilitates early weaning from tracheotomy in children. Pediatr Crit Care Med.

